# Potential Role of DEC1 in Cervical Cancer Cells Involving Overexpression and Apoptosis

**DOI:** 10.3390/clockssleep2010004

**Published:** 2020-01-26

**Authors:** Fuyuki Sato, Ujjal K. Bhawal, Nao Sugiyama, Shoko Osaki, Kosuke Oikawa, Yasuteru Muragaki

**Affiliations:** 1 Department of Pathology, Wakayama Medical University School of Medicine, Wakayama 641-8509, Japan; d1666040@wakayama-med.ac.jp (N.S.); osakipon@gmail.com (S.O.); k-oikawa@wakayama-med.ac.jp (K.O.); ymuragak@wakayama-med.ac.jp (Y.M.); 2 Department of Diagnostic Pathology, Shizuoka Cancer Center, Sunto-gun 411-8777, Japan; 3 Department of Biochemistry and Molecular Biology, Nihon University School of Dentistry at Matsudo, Chiba 271-8587, Japan; bhawal.ujjal.kumar@nihon-u.ac.jp

**Keywords:** DEC1, SOX2, c-MYC, immunohistochemistry, cervical cancer

## Abstract

Basic helix-loop-helix (BHLH) transcription factors differentiated embryonic chondrocyte gene 1 (DEC1) and gene 2 (DEC2) regulate circadian rhythms, apoptosis, epithelial mesenchymal transition (EMT), invasions and metastases in various kinds of cancer. The stem cell markers SOX2 and c-MYC are involved in the regulation of apoptosis and poor prognosis. In cervical cancer, however, their roles are not well elucidated yet. To determine the function of these genes in human cervical cancer, we examined the expression of DEC1, DEC2, SOX2 and c-MYC in human cervical cancer tissues. In immunohistochemistry, they were strongly expressed in cancer cells compared with in non-cancerous cells. Notably, the strong rate of DEC1 and SOX2 expressions were over 80% among 20 cases. We further examined the roles of DEC1 and DEC2 in apoptosis. Human cervical cancer HeLa and SiHa cells were treated with cisplatin—HeLa cells were sensitive to apoptosis, but SiHa cells were resistant. DEC1 expression decreased in the cisplatin-treated HeLa cells, but had little effect on SiHa cells. Combination treatment of DEC1 overexpression and cisplatin inhibited apoptosis and affected SOX2 and c-MYC expressions in HeLa cells. Meanwhile, DEC2 overexpression had little effect on apoptosis and on SOX2 and c-MYC expressions. We conclude that DEC1 has anti-apoptotic effects and regulates SOX2 and c-MYC expressions on apoptosis.

## 1. Introduction

It has been shown that the basic helix-loop-helix (BHLH) transcription factor differentiated embryonic chondrocyte gene 1 (DEC1/BHLHE40/Stra13) is strongly expressed in oral, thyroid, breast, gastric and pancreatic cancers and in hepatocellular carcinoma cells compared to non-cancerous cells. It involves epithelial mesenchymal transition (EMT), recurrence and poor prognosis [1,2,3,4,5,6,7,8]. On the other hand, DEC2 (BHLHE41) expression varies between different types of cancer. DEC2 is more strongly expressed in thyroid cancer cells and carcinoma cells of osteosarcoma than in non-cancerous cells, but weakly expressed in gastric and pancreatic cancer cells [4,9,10,11]. We showed that DEC2 has circadian expression in tumor cells, such as in mouse sarcoma and in human hepatocellular carcinoma, osteosarcoma and breast cancer cells [12,13,14]. We have also shown that DEC1 and DEC2 play important roles in the regulation of apoptosis in human breast and oral cancer [1,15,16,17]. Others have also shown that DEC1 and DEC2 regulate apoptosis in various kinds of cancer [18,19,20,21,22]. DEC1 and DEC2 have pro- or anti-apoptotic effects and their functions differ between tissues [1]. DEC1 has pro-apoptotic effects in breast cancer, for example, but anti-apoptotic effects in gastric and colon cancers [17,18,23]. DEC1 function on apoptosis may therefore vary between different cancer tissues. In cervical cancer, the significant roles of DEC1 and DEC2 are still not well understood.

Cisplatin is one of the standard chemotherapy treatments against cervical cancer, but resistance to it may prevent effective treatment and advance tumor progression [24,25]. Abnormalities of Bcl2 and p53 expressions and caspases activity are associated with cisplatin resistance [24]. The stem cell markers c-MYC and SOX2 play important roles in the regulation of differentiation and also regulate cancer cell growth and chemotherapy resistance [26,27]. SOX2 regulates radio-resistance in cervical cancer [28]. Increases in vimentin are associated with EMT, metastasis and poor prognosis [7,29]. Meanwhile, the molecular mechanisms of chemotherapy resistance in cervical cancer are not yet fully understood in detail. In this study, we examine DEC1 and DEC2 expressions in cervical cancer tissues and apoptotic reaction to cisplatin treatment.

## 2. Results

### 2.1. DEC1 and DEC2 Expressions in Cervical Cancer Tissues

**Figure 1:**
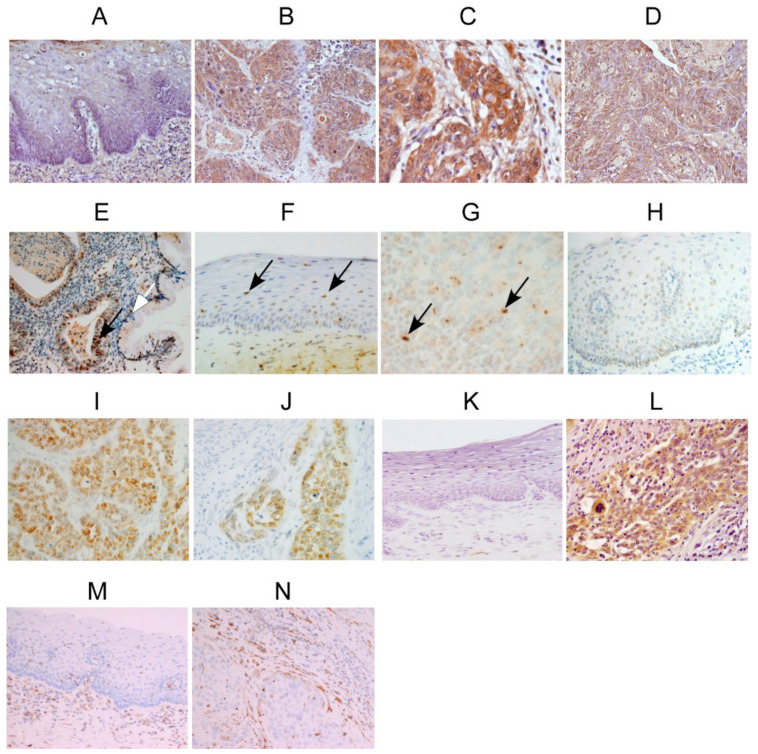
Immunoreactivities of DEC1, DEC2, SOX2, c-MYC and vimentin in cervical cancer tissues. Representative images for the immunoreactivities of DEC1, DEC2, SOX2, c-MYC and vimentin in cervical cancer tissues. Each panel shows ×200 magnification. DEC1 immunoreactivities in (**A**) non-cancerous cells, (**B**) shallow cancer cells, and (**C**) deep cancer cells of case 14. DEC1 immunoreactivity in (**D**) SCC of case 1, and in (**E**) AC of case 5, respectively. DEC2 immunoreactivities in (**F**) non-cancerous cells, (**G**) and cancer cells of case 15. SOX2 immunoreactivities in (**H**) non-cancerous cells, (**I**) shallow cancer cells, and (**J**) deep cancer cells of case 14. c-MYC immunoreactivities in (**K**) non-cancerous cells, (**L**) and cancer cells of case 15. Vimentin immunoreactivities in (**M**) non-cancerous vascular cells, (**N**) and cancerous vascular cells of case 14. Black arrows in E-G show nuclear staining. The white arrow in E indicates non-cancerous cells.

Representative images of DEC1, DEC2, SOX2, c-MYC and vimentin immunoreactivities of cervical cancer tissues are shown in Figure 1. In surgical resection specimens, DEC1 was strongly detected in the cytoplasm and nucleus of cancer cells, but was weakly detected in non-cancerous cells (Figure 1A,B). It was more strongly stained in the deep invasive cancer cells than in the shallow cancer cells (Figure 1C). In biopsy specimens, DEC1 was strongly expressed in cancer cells of both squamous cell carcinoma and adenocarcinoma (Figure 1D,E). In a resection specimen, DEC2 was weakly detected in the nucleus of non-cancerous cells and moderately stained in cancer cells, respectively (Figure 1F,G). SOX2 was barely detected in non-cancerous cells, but strongly expressed in the nucleus of cancer cells (Figure 1H,I). No obvious differences were observed between shallow and deep invasive cancer cells (Figure 1J). c-MYC was also barely detected in non-cancerous cells, but moderately stained in the cytoplasm of cancer cells (Figure 1K,L). Vimentin was barely detected in non-cancerous vascular cells, but strongly expressed in cancerous vascular cells (Figure 1M,N). We summarize their immunoreactivities in Table 1, Table 2, Table 3 and Table 4.

### 2.2. DEC1 Expression Decreased in Apoptosis-Induced HeLa Cells

**Figure 2:**
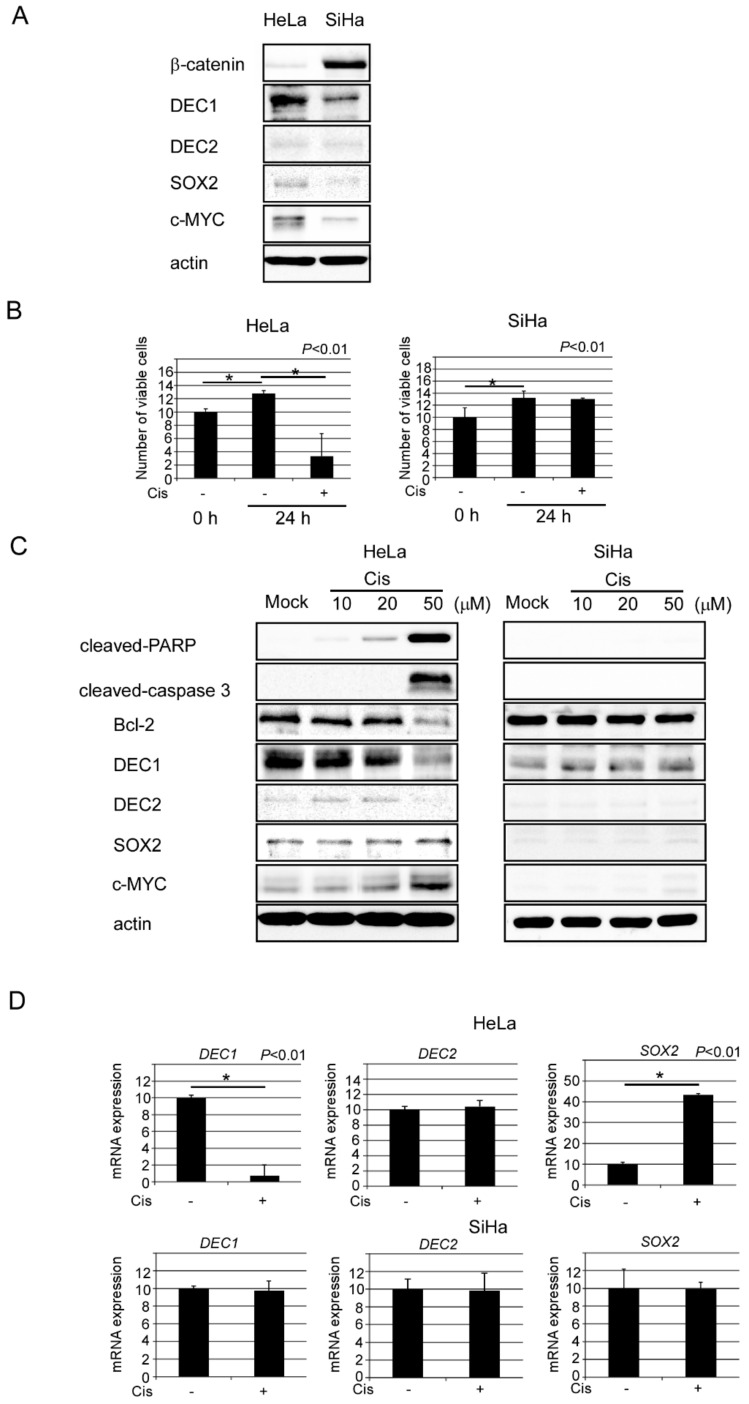
DEC1 expression decreases in HeLa cells under apoptosis. (**A**) Endogenous DEC1 protein expressions in HeLa and SiHa cells. Western blotting images of *β*-catenin, DEC1, DEC2, SOX2, c-MYC and actin in HeLa and SiHa cells. The western blot analysis was repeated three times and similar results were obtained. (**B**) Cell viability of HeLa and SiHa cells with (+) or without (−) cisplatin treatment was determined. The absorbance (OD_480_/OD_650_) at 0 and 24 h is shown. The value of cell viability of control 0 h regarded as 10, which means each basal value without treatment. Data are expressed as mean values ± SE (bars) of three independent samples. * *p* <0.01, as determined using Dunnett’s test. Cis: Cisplatin treatment. (**C**) Western blotting images of cleaved-poly (ADP-ribose) polymerase (PARP), cleaved-caspase 3, Bcl-2, DEC1, DEC2, SOX2, c-MYC and actin treated with 10, 20, and 50 μM cisplatin or mock in HeLa and SiHa cells. The western blot analysis was repeated three times and similar results were obtained (**D**) *DEC1*, *DEC2* and *SOX2* mRNA expressions in HeLa and SiHa cells with (+) or without (−) cisplatin treatment. Data are expressed as mean values ± SE (bars) of three independent samples. * *p* < 0.01, as determined using t-test. qPCR was repeated three times and similar results were obtained.

To investigate the functions of DEC1 and DEC2 on apoptosis, we first examined the endogenous protein levels of DEC1 and DEC2 by the western blotting of human cervical cancer HeLa and SiHa cells (Figure 2A). DEC1 protein expression was strongly detected in HeLa cells, but weakly in SiHa cells. SOX2 and c-MYC protein expressions were slightly detected in HeLa cells, but they had little detection in SiHa cells. On the other hand, while *β*-catenin protein expression was strongly detected in SiHa cells, it was barely detected in HeLa cells. Endogenous DEC2 protein expression had little detection in both HeLa and SiHa cells. Next, we investigated whether DEC1 and DEC2 expressions were affected by cisplatin-induced apoptosis, again using HeLa and SiHa cells. HeLa cells were sensitive to apoptosis, SiHa cells were resistant. As expected, cisplatin treatment for 24 h decreased the cell viability of HeLa, but had little effect on the cell viability of SiHa (Figure 2B). To examine the DEC1 and DEC2 expressions by apoptosis, we treated cells with cisplatin in a concentration-dependent manner, and performed western blotting. The protein amounts of apoptotic markers cleaved-poly (ADP-ribose) polymerase (PARP) and caspase 3 were significantly increased in 50 μM cisplatin treatment of HeLa cells (Figure 2C). Another apoptotic marker, Bcl-2, decreased in 50 μM of cisplatin treatment in HeLa cells. The optimal concentration of cisplatin is, therefore, 50 μM for further apoptosis analysis. DEC1 expression significantly decreased in 50 μM of cisplatin treatment in HeLa cells, but DEC2 expression was barely affected. SOX2 expression slightly increased in 50 μM of cisplatin treatment, c-MYC expression significantly increased. Since SiHa cells are resistant to apoptosis, these apoptotic markers were barely affected by cisplatin treatment. Expressions of DEC1, DEC2, SOX2 and c-MYC in SiHa cells were also barely affected. In real-time PCR analysis, the mRNA expressions of *DEC1*, *DEC2* and *SOX2* by cisplatin treatment gave similar results to their protein levels (Figure 2D). 

### 2.3. DEC1 Overexpression Antagonizes Cisplatin-Induced Apoptosis

**Figure 3:**
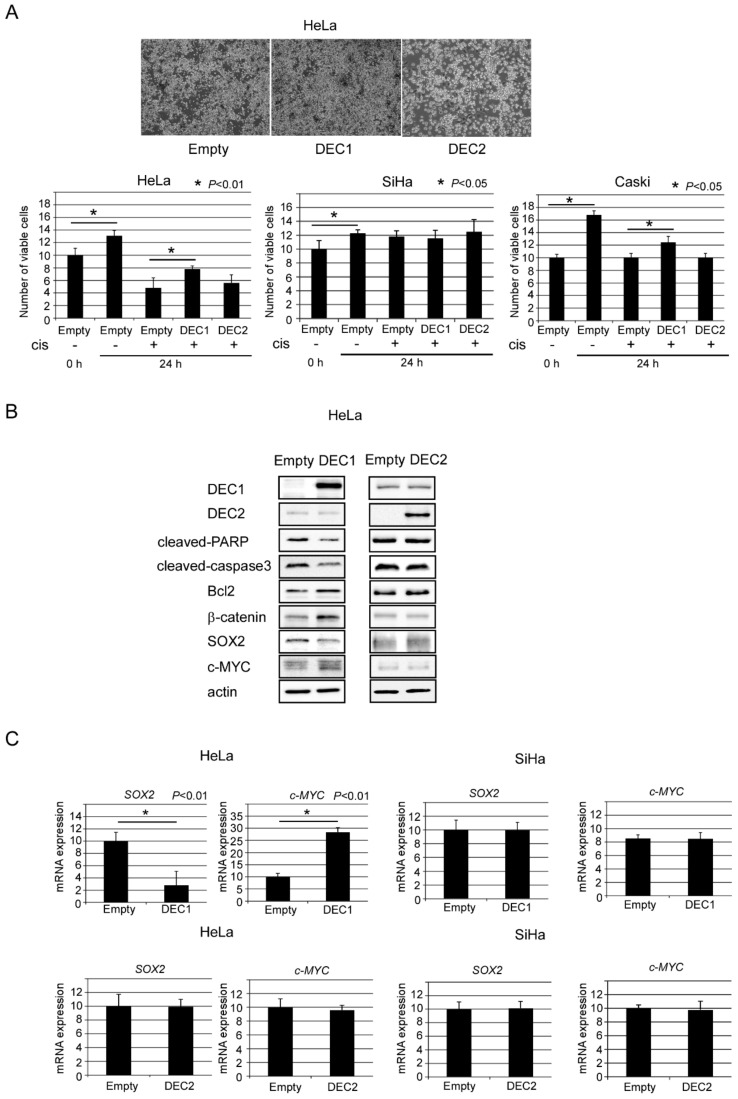
DEC1 has anti-apoptotic effects in HeLa cells under apoptosis. (**A**) Cell viability of HeLa, SiHa and Caski cells with (+) or without (−) cisplatin and empty vector, DEC1 or DEC2 plasmid treatment was determined. The absorbance (OD_480_/OD_650_) at 0 and 24 h is shown. The value of cell viability of control 0 h regarded as 10. Data are expressed as mean values ± SE (bars) of three independent samples. * *p* < 0.05, as determined using *t*-test. (**B**) Western blotting images of DEC1, DEC2, cleaved-PARP, cleaved-caspase 3, Bcl-2, *β*-catenin, SOX2, c-MYC and actin treated with 50 μM cisplatin and empty vector, DEC1 or DEC2 plasmid in HeLa cells. Western blot analysis was repeated three times and similar results were obtained. (**C**) *SOX2* and *c-MYC* mRNA expressions in HeLa and SiHa cells with cisplatin and empty vector, DEC1 or DEC2 plasmid treatment. Data are expressed as mean values ± SE (bars) of three independent samples. * *p* < 0.01, as determined using t-test. qPCR was repeated three times and similar results were obtained.

We further examined the effect of DEC1 and DEC2 on apoptosis using plasmid transfection. The empty vector introduction with cisplatin treatment visually induced cell death of HeLa (Figure 3A top panel). Compared to the control, DEC1 overexpression with cisplatin treatment inhibited cell death, whereas DEC2 overexpression had little effect on the cell death. We quantified cell viability of HeLa, SiHa and another apoptosis-sensitive cervical cancer cell, Caski, by combination treatment of DEC1 or DEC2 overexpression and cisplatin (Figure 3A bottom panel). DEC1 overexpression increased cell viability of HeLa and Caski, but DEC2 overexpression had little effect. Neither DEC1 nor DEC2 overexpression with cisplatin treatment had much effect on the cell viability of SiHa. These results suggest that DEC1 overexpression inhibited cell death induced by cisplatin treatment. We then analyzed the apoptotic markers, *β*-catenin, SOX2 and c-MYC expressions for how they were affected by DEC1 or DEC2 overexpression. DEC1 overexpression with cisplatin treatment decreased the amounts of cleaved-PARP, caspase 3 and SOX2, but it increased the amounts of Bcl2, *β*-catenin and c-MYC (Figure 3B). On the other hand, DEC2 overexpression had little effect on their amounts. In real time PCR analysis, similar results of *SOX2* and *c-MYC* as the protein levels were observed (Figure 3C). 

## 3. Discussion

Although it is well proven that DEC1 is strongly expressed in various kinds of cancer cell, its expression in cervical cancer tissues is still unknown [1,2,3,4,5,6,7,8]. We showed that DEC1 is strongly expressed in cervical cancer cells and the ratio was 85% among 20 cases. This strongly suggests that DEC1 is closely associated with tumor progression in the cervix. We also found that the DEC1 expression was higher in deep invasive cancer cells than shallow cancer cells in cases 13, 14 and 19. Taken together, DEC1 may be associated with EMT, as in pancreatic cancer [7]. Since transforming growth factor- beta (TGF-β), SMAD3 and hypoxia inducible factor 1 alpha (HIF-1α) are upstream factors of DEC1 [1], the DEC1 increases in cervical cancer cells may depend on their function under hypoxia and inflammation. DEC2 was also strongly detected in cervical cancer cells, although the strong ratio was less than that of DEC1. DEC2 was dominantly expressed in the nucleus of cancer cells, especially mitotic cells, suggesting that DEC2 also works in tumor progression in the cervix. Conversely, DEC2 overexpression had little effect on apoptosis. We therefore speculate that DEC2 may be involved in other functions, such as the regulation of cell cycle. 

It has been reported that both SOX2 and c-MYC have anti-apoptotic effects in gastric, ovarian, head and neck and thyroid cancer cells [30,31,32,33]. SOX2 expression was increased and decreased on apoptosis, and the alternation may depend on differential tissues and apoptosis inducer [30,31,32]. We revealed that DEC1 overexpression on apoptosis decreased the expression of SOX2, but it increased the expression of c-MYC. These findings suggest that SOX2 may have pro-apoptotic effects, whereas c-MYC has anti-apoptotic effetcs. Luo J et al. demonstrated that SOX2 overexpression induced cell cycle arrest and apoptosis in gastric cancer cells [30]. This observation is compatible with our findings. We showed that the strong ratio of SOX2 expression in cervical cancer cells was 80%, but the ratio of c-MYC was 35%. This difference may be associated with the differential regulation by DEC1 overexpression. Notably, DEC1 affected the SOX2 and c-MYC expressions in HeLa, but not those in SiHa cells. This implies that DEC1 regulates them on apoptosis. A previous report showed that *β*-catenin might be a regulator of stem cell markers and could affect the expression of SOX2, c-MYC and vimentin in nasopharyngeal carcinoma cells [34]. The upregulation of *β*-catenin induces cell proliferation and resistance to apoptosis [35]. We found DEC1 overexpression increased *β*-catenin expression on apoptosis. These results imply that DEC1 has anti-apoptotic effects via *β*-catenin induction. In addition to this, another group showed that SOX2 regulates c-MYC expression [36]. Collectively, it is possible that DEC1 regulates SOX2 and c-MYC expressions via *β*-catenin under apoptosis. 

In summary, we suggest that DEC1 has anti-apoptotic effects via SOX2 and c-MYC. Collectively, DEC1, DEC2, SOX2 and c-MYC play important roles of cervical cancer progression. DEC1 may be a candidate upstream factor of stem cell markers on apoptosis.

## 4. Materials and Methods

### 4.1. Tissue Preparation 

Human biopsies and surgical specimens of cervical cancer from between January 2011 and December 2017 were obtained from the Department of Diagnostic Pathology, Wakayama Medical University. Diagnosis was performed by at least two pathologists. Clinical and pathological information is summarized in Table 1.

### 4.2. Ethics Approval and Consent to Participate

This study was approved by the Wakayama Medical University Research Ethics Committee (15 December 2015, Protocol No. 1715) and histological specimens were retrieved from our hospital archives.

### 4.3. Immunohistochemistry 

Ten 4 μm serial sections were prepared for staining and incubated with primary antibodies for 2 h. Immunohistochemistry was performed using a Discovery Auto-Stainer with automated protocols (Ventana Medical Systems, Inc., Tucson, AZ, USA; Roche, Mannheim, Germany) as previously described [37,38]. The intensities of DEC1, DEC2, SOX2, c-MYC and vimentin were determined by qualitative assessment of three levels: weak (1), moderate (2) and strong (3), as previously described [38].

### 4.4. Cell Culture and Treatment

HeLa, SiHa and Caski human cervical cancer cells were obtained from the American Type Culture Collection (ATCC; Manassa, VA, USA). These cells were cultured in Dulbecco’s modified Eagle’s medium (DMEM) (Sigma Chemical Co., St. Louis, MO, USA) supplemented with 10% fetal bovine serum and 1% antibiotics. Transient plasmid transfection of DEC1 and DEC2 was performed as previously described [37]. Cisplatin (Sigma) treatment at various concentrations for 24 h was performed as previously described [39].

### 4.5. Western Blot

The cells were lysed using M-PER protein lysis buffer (Thermo Fisher Scientific, Inc.). Protein determination was performed using the bicinchoninic method [38], and the 40 µg proteins of the total cell lysates were run on SDS-polyacrylamide gels followed by western blotting using standard procedures. A WesternBright Sirius Kit (Advansta, CA, USA) was used for antibody detection, and an AE-9300 Ez capture MG (ATTO, Tokyo, Japan) was used for image data capture. We repeated the western blot analysis three times and the results were similar.

### 4.6. Antibodies

The following commercial antibodies were purchased: *β*-catenin (1:10000, rabbit polyclonal, sc-7199; Santa Cruz Biotechnology Inc., Santa Cruz, CA, USA), DEC1 (1:1000, rabbit polyclonal, NB100-1800; Novus Biologicals), DEC2 (1:200, mouse monoclonal, sc-373763; Santa Cruz Biotechnology Inc), SOX2 (1:1000, rabbit polyclonal, Ab97959, Abcam), c-MYC (1:2000, rabbit monoclonal, Ab32072, Abcam), vimentin (1:3000, rabbit monoclonal, Ab92547, Abcam), cleaved-PARP (1:1000, rabbit polyclonal, Cell Signaling Technology, Inc., Danvers, MA, USA), cleaved-caspase3 (1:500, rabbit polyclonal, Cell Signaling Technology, Inc), Bcl-2 (1:500, rabbit polyclonal, sc-492; Santa Cruz Biotechnology Inc), and actin (1:10000, mouse monoclonal, A5441; Sigma Chemical Co.). 

### 4.7. Real-Time (Quantitative) PCR (qPCR)

We prepared three independent RNA samples from HeLa and SiHa cells. The total RNA was isolated and first-strand cDNA was synthesized as previously described [38]. Real-time PCR was performed using SYBR Green Master Mix (Bio-Rad Laboratories, Inc., Hercules, CA, USA). The amplification primer sequences were designed as follows: *DEC1*-F, 5′-GAAAGGATCGGCGCAATTAA-3′ and R, 5′-CATCATCCGAAAGCTGCATC-3′; *DEC2*-F, 5′-CGCCCATTCAGTCCGACTT-3′ and R, 5′-CGGGAGAGGTATTGCAAGACTT-3′; *SOX2*-F, 5′-GAATGCCTTCATGGTGTGGT-3′ and R, 5′-TTGCTGATCTCCGAGTTGTG-3′; *c-MYC*-F, 5′-CGTCTCCACACATCAGCACAA-3′ and R, 5′-TCTTGGCAGCAGGATAGTCCTT-3′; and *18S* rRNA-F, 5′-GCGCCGCTAGAGGTGAAAT-3′ and R, 5′- GAAAACATTCTTGGCAAATGCTT-3′. The data were normalized using *18S* rRNA. qPCR was repeated three times and the results were similar. The 2^−∆∆Cq^ method was used for relative quantification [40].

### 4.8. Cell Viability Assay

The cell viability assay was performed using XTT kit (XTT-based) (Biological Industries). Cells were seeded into 96-well plates. XTT kit reaction solution was added to each well and the cells were incubated at 37 °C for an additional 2 h according to the manufacturer’s instructions. Absorbance at an optical density at 480 nm (OD_480_) and at (OD_650_) was measured using a 96-well microplate reader (SH-9000; Hitachi). Bright field pictures after treatment with cisplatin and plasmid transfection were taken using a microscopic digital camera (Nikon DS-L3; Nikon Corporation, Tokyo, Japan). 

## Figures and Tables

**Table 1:** Immunohistochemical detection of differentiated embryonic chondrocyte gene 1 (DEC1) proteins in human cervical cancer tissues.

DEC1					
C	M	A	PD	T	N
1	Biopsy	73	SCC	Strong	NI
2	Biopsy	57	SCC	Strong	NI
3	Biopsy	50	SCC	Strong	NI
4	Biopsy	52	AC	Strong	NI
5	Biopsy	44	AC	Strong	Weak
6	Biopsy	54	SCC	Strong	Weak
7	Biopsy	72	SCC	Strong	NI
8	Biopsy	78	SCC	Strong	NI
9	Biopsy	45	SCC	Strong	Weak
10	Biopsy	57	SCC	Strong	NI
11	Biopsy	41	SCC	Strong	NI
12	Biopsy	27	SCC	Strong	NI
13	Resection	46	SCC	Strong	Weak
14	Resection	38	SCC	Strong	Weak
15	Resection	35	SCC	Strong	Weak
16	Resection	44	SCC	Strong	Weak
17	Resection	37	SCC	Weak	Moderate
18	Resection	38	SCC	Moderate	Weak
19	Resection	82	SCC	Strong	Weak
20	Resection	48	SCC	Moderate	Weak

C, cases; M, materials; A, age; PD, pathological diagnosis; T, tumor cells; N, adjacent non-tumor cells; SCC, squamous cell carcinoma; AC, adenocarcinoma; NI, not included of non-tumor cells.

**Table 2:** Immunohistochemical detection of DEC2, SOX2 and c-MYC proteins in human cervical cancer tissues.

	DEC2		SOX2		c-MYC	
C	T	N	T	N	T	N
1	Strong	NI	Strong	NI	Moderate	NI
2	Strong	NI	Strong	NI	Moderate	NI
3	Moderate	NI	Strong	NI	Weak	NI
4	Strong	NI	Strong	NI	Weak	NI
5	Strong	Strong	Weak	Weak	Weak	Weak
6	Strong	Weak	Strong	Weak	Weak	Weak
7	Strong	NI	Strong	NI	Strong	NI
8	Strong	NI	Strong	NI	Moderate	NI
9	Moderate	Moderate	Strong	Weak	Strong	Weak
10	Strong	NI	Strong	NI	Strong	NI
11	Strong	NI	Strong	NI	Strong	NI
12	Strong	NI	Strong	NI	Moderate	NI
13	Strong	Weak	Strong	Weak	Moderate	NI
14	Moderate	Weak	Strong	Weak	Weak	Weak
15	Strong	Weak	Strong	Weak	Moderate	Weak
16	Weak	Weak	Strong	Weak	Weak	Weak
17	Moderate	Weak	Weak	Strong	Strong	Weak
18	Weak	Weak	Moderate	Moderate	Weak	Weak
19	Moderate	Weak	Weak	Weak	Strong	Weak
20	Weak	Weak	Strong	Weak	Strong	Weak

C, cases; T, tumor cells; N, adjacent non-tumor cells; NI, not included of non-tumor cells.

**Table 3:** Immunohistochemical detection of vimentin proteins in human cervical cancer tissues.

Vimentin		
C	T	N
1	Strong	NI
2	Strong	NI
3	Strong	NI
4	Strong	NI
5	Strong	Weak
6	Strong	Weak
7	Strong	NI
8	Strong	NI
9	Strong	Moderate
10	Strong	NI
11	Strong	NI
12	Strong	NI
13	Strong	Weak
14	Strong	Weak
15	Strong	Weak
16	Moderate	Weak
17	Strong	Weak
18	Strong	Moderate
19	Strong	Weak
20	Moderate	Weak

C, cases; T, tumor cells; N, adjacent non-tumor cells; NI, not included of non-tumor cells.

**Table 4:** Summary of immunohistochemical detection in tumor lesions.

	DEC1	DEC2	SOX2	c-MYC	Vimentin
Strong	85%	60%	80%	35%	90%
Moderate	10%	25%	5%	30%	10%
Weak	5%	15%	15%	35%	0%

## Abbreviations

*DEC1*	*Differentiated embryonic chondrocyte gene 1*
*DEC2*	*Differentiated embryonic chondrocyte gene 2*
*SOX2*	*SRY-box2*

## References

[1] Sato F., Bhawal U.K., Yoshimura T., Muragaki Y. (2016). DEC1 and DEC2 Crosstalk Between Circadian Rhythm and Tumor Progression. J. Cancer.

[2] You J., Lin L., Liu Q., Zhu T., Xia K., Su T. (2014). The correlation between theexpression of differentiated embryo-chondrocyte expressed gene l and oral squamous cell carcinoma. Eur. J. Med. Res..

[3] Bhawal U.K., Sato F., Arakawa Y., Fujimoto K., Kawamoto T., Tanimoto K., Ito Y., Sasahira T., Sakurai T., Kobayashi M. (2011). Basic helix-loop-helix transcription factor DEC1 negatively regulates cyclin D1. J. Pathol..

[4] Gallo C., Fragliasso V., Donati B., Torricelli F., Tameni A., Piana S., Ciarrocchi A. (2018). The bHLH transcription factor DEC1 promotes thyroid cancer aggressiveness by the interplay with NOTCH1. Cell Death Dis..

[5] Liu Y., Miao Y., Wang J., Lin X., Wang L., Xu H.T., Wang E.H. (2013). DEC1 is positively associated with the malignant phenotype of invasive breast cancers and negatively correlated with the expression of claudin-1. Int. J. Mol. Med..

[6] Jia Y.F., Xiao D.J., Ma X.L., Song Y.Y., Hu R., Kong Y., Zheng Y., Han S.Y., Hong R.L., Wang Y.S. (2013). Differentiated embryonic chondrocyte-expressed gene 1 is associated with hypoxia-inducible factor 1α and Ki67 in human gastric cancer. Diagn. Pathol..

[7] Wu Y., Sato F., Yamada T., Bhawal U.K., Kawamoto T., Fujimoto K., Noshiro M., Seino H., Morohashi S., Hakamada K. (2012). The BHLH transcription factor DEC1 plays an important role in the epithelial-mesenchymal transition of pancreatic cancer. Int. J. Oncol..

[8] Shi X.H., Zheng Y., Sun Q., Cui J., Liu Q.H., Qü F., Wang Y.S. (2011). DEC1 nuclear expression: A marker of differentiation grade in hepatocellular carcinoma. World J. Gastroenterol..

[9] Hu T., He N., Yang Y., Yin C., Sang N., Yang Q. (2015). DEC2 expression is positively correlated with HIF-1 activation and the invasiveness of human osteosarcomas. J. Exp. Clin. Cancer Res..

[10] Li P., Jia Y.F., Ma X.L., Zheng Y., Kong Y., Zhang Y., Zong S., Chen Z.T., Wang Y.S. (2016). DEC2 suppresses tumor proliferation and metastasis by regulating ERK/NF-κB pathway in gastric cancer. Am. J. Cancer Res..

[11] Sato F., Kawamura H., Wu Y., Sato H., Jin D., Bhawal U.K., Kawamoto T., Fujimoto K., Noshiro M., Seino H. (2012). The basic helix-loop-helix transcription factor DEC2 inhibits TGF-β-induced tumor progression in human pancreatic cancer BxPC-3 cells. Int. J. Mol. Med..

[12] Sato F., Bhawal U.K., Kawamoto T., Fujimoto K., Imaizumi T., Imanaka T., Kondo J., Koyanagi S., Noshiro M., Yoshida H. (2008). Basic-helix-loop-helix (bHLH) transcription factor DEC2 negatively regulates vascular endothelial growth factor expression. Genes Cells.

[13] Matsunaga N., Inoue M., Kusunose N., Kakimoto K., Hamamura K., Hanada Y., Toi A., Yoshiyama Y., Sato F., Fujimoto K. (2012). Time-dependent interaction between differentiated embryo chondrocyte-2 and CCAAT/enhancer-binding protein α underlies the circadian expression of CYP2D6 in serum-shocked HepG2 cells. Mol. Pharmacol..

[14] Sato F., Muragaki Y., Kawamoto T., Fujimoto K., Kato Y., Zhang Y. (2016). Rhythmic expression of DEC2 protein in vitro and in vivo. Biomed. Rep..

[15] Liu Y., Sato F., Kawamoto T., Fujimoto K., Morohashi S., Akasaka H., Kondo J., Wu Y., Noshiro M., Kato Y. (2010). Anti-apoptotic effect of the basic helix-loop-helix (bHLH) transcription factor DEC2 in human breast cancer cells. Genes Cells.

[16] Wu Y., Sato F., Bhawal U.K., Kawamoto T., Fujimoto K., Noshiro M., Seino H., Morohashi S., Kato Y., Kijima H. (2012). BHLH transcription factor DEC2 regulates pro-apoptotic factor Bim in human oral cancer HSC-3 cells. Biomed. Res..

[17] Wu Y., Sato F., Bhawal U.K., Kawamoto T., Fujimoto K., Noshiro M., Morohashi S., Kato Y., Kijima H. (2011). Basic helix-loop-helix transcription factors DEC1 and DEC2 regulate the paclitaxel-induced apoptotic pathway of MCF-7 human breast cancer cells. Int. J. Mol. Med..

[18] Jia Y., Hu R., Li P., Zheng Y., Wang Y., Ma X. (2018). DEC1 is required for anti-apoptotic activity of gastric cancer cells under hypoxia by promoting Survivin expression. Gastric Cancer.

[19] Li X.M., Lin W., Wang J., Zhang W., Yin A.A., Huang Y., Zhang J., Yao L., Bian H., Zhang J. (2016). Dec1 expression predicts prognosis and the response to temozolomide chemotherapy in patients with glioma. Mol. Med. Rep..

[20] Peng Y., Liu W., Xiong J., Gui H.Y., Feng X.M., Chen R.N., Hu G., Yang J. (2012). Down regulation of differentiated embryonic chondrocytes 1 (DEC1) is involved in 8-methoxypsoralen-induced apoptosis in HepG2 cells. Toxicology.

[21] Li H., Ma X., Xiao D., Jia Y., Wang Y. (2018). Expression of DEC2 enhances chemosensitivity by inhibiting STAT5A in gastric cancer. J. Cell Biochem..

[22] Jiang B., Mu W., Wang J., Lu J., Jiang S., Li L., Xu H., Tian H. (2016). MicroRNA-138 functions as a tumor suppressor in osteosarcoma by targeting differentiated embryonic chondrocyte gene 2. J. Exp. Clin. Cancer Res..

[23] Li Y., Zhang H., Xie M., Hu M., Ge S., Yang D., Wan Y., Yan B. (2002). Abundant expression of Dec1/stra13/sharp2 in colon carcinoma: Its antagonizing role in serum deprivation-induced apoptosis and selective inhibition of procaspase activation. Biochem. J..

[24] Zhu H., Luo H., Zhang W., Shen Z., Hu X., Zhu X. (2016). Molecular mechanisms of cisplatin resistance in cervical cancer. Drug Des. Devel Ther..

[25] Pectasides D., Kamposioras K., Papaxoinis G., Pectasides E. (2008). Chemotherapy for recurrent cervical cancer. Cancer Treat. Rev..

[26] Zhen H.Y., He Q.H., Zhen Y.Z., Wang S.L., Liu Y.N., Wu W.H., Zhang X.Y., Lu A.L., Shen L. (2011). Inhibition of mouse embryonic carcinoma cell growth by lidamycin through down-regulation of embryonic stem cell-like genes Oct4, Sox2 and Myc. Investig. New Drugs.

[27] Cheng C.C., Shi L.H., Wang X.J., Wang S.X., Wan X.Q., Liu S.R., Wang Y.F., Lu Z., Wang L.H., Ding Y. (2018). Stat3/Oct-4/c-Myc signal circuit for regulating stemness-mediated doxorubicin resistance of triple-negative breast cancer cells and inhibitory effects of WP1066. Int. J. Oncol..

[28] Huang C., Lu H., Li J., Xie X., Fan L., Wang D., Tan W., Wang Y., Lin Z., Yao T. (2018). SOX2 regulates radioresistance in cervical cancer via the hedgehog signaling pathway. Gynecol. Oncol..

[29] Lin J., Lu J., Wang C., Xue X. (2017). The prognostic values of the expression of Vimentin, TP53, and Podoplanin in patients with cervical cancer. Cancer Cell Int..

[30] Luo J., Yan R., He X., He J. (2018). SOX2 inhibits cell proliferation and metastasis, promotes apoptotic by downregulating CCND1 and PARP in gastric cancer. Am. J. Transl. Res..

[31] Li Y., Chen K., Li L., Li R., Zhang J., Ren W. (2015). Overexpression of SOX2 is involved in paclitaxel resistance of ovarian cancer via the PI3K/Akt pathway. Tumour Biol..

[32] Schröck A., Bode M., Göke F.J., Bareiss P.M., Schairer R., Wang H., Weichert W., Franzen A., Kirsten R., van Bremen T. (2014). Expression and role of the embryonic protein SOX2 in head and neck squamous cell carcinoma. Carcinogenesis.

[33] Kim S.H., Kang J.G., Kim C.S., Ihm S.H., Choi M.G., Yoo H.J., Lee S.J. (2013). Apigenin induces c-Myc-mediated apoptosis in FRO anaplastic thyroid carcinoma cells. Mol. Cell Endocrinol..

[34] Jiang R., Niu X., Huang Y., Wang X. (2016). β-Catenin is important for cancer stem cellgeneration and tumorigenic activity in nasopharyngeal carcinoma. Biochim. Biophys. Sin. (Shanghai).

[35] Thévenod F., Chakrabortym P.K. (2010). The role of Wnt/beta-catenin signaling in renal carcinogenesis: Lessons from cadmium toxicity studies. Curr. Mol. Med..

[36] Chen S., Xu Y., Chen Y., Li X., Mou W., Wang L., Liu Y., Reisfeld R.A., Xiang R., Lv D. (2012). SOX2 gene regulates the transcriptional network of oncogenes and affects tumorigenesis of human lung cancer cells. PLoS ONE.

[37] Sato F., Otsuka T., Kohsaka A., Le H.T., Bhawal U.K., Muragaki Y. (2019). Smad3 Suppresses Epithelial Cell Migration and Proliferation via the Clock Gene Dec1, Which Negatively Regulates the Expression of Clock Genes Dec2 and Per1. Am. J. Pathol..

[38] Sato F., Bhawal U.K., Tojyo I., Fujita S., Murata S.I., Muragaki Y. (2019). Differential expression of claudin-4, occludin, SOX2 and proliferating cell nuclear antigen between basaloid squamous cell carcinoma and squamous cell carcinoma. Mol. Med. Rep..

[39] Sato F., Wu Y., Bhawal U.K., Liu Y., Imaizumi T., Morohashi S., Kato Y., Kijima H. (2011). PERIOD1 (PER1) has anti-apoptotic effects, and PER3 has pro-apoptotic effects during cisplatin (CDDP) treatment in human gingival cancer CA9-22 cells. Eur. J. Cancer.

[40] Livak K.J., Schmittgen T.D. (2001). Analysis of relative gene expression data using real-time quantitative PCR and the 2(-Delta Delta C(T)) Method. Methods.

